# Establishment of P38Bf, a Core-Fucose-Deficient Mouse-Canine Chimeric Antibody Against Dog Podoplanin

**DOI:** 10.1089/mab.2018.0035

**Published:** 2018-10-24

**Authors:** Yukinari Kato, Takuya Mizuno, Shinji Yamada, Takuro Nakamura, Shunsuke Itai, Miyuki Yanaka, Masato Sano, Mika K. Kaneko

**Affiliations:** ^1^ Department of Antibody Drug Development, Tohoku University Graduate School of Medicine, Sendai, Japan.; ^2^ New Industry Creation Hatchery Center, Tohoku University, Sendai, Japan.; ^3^ Laboratory of Molecular Diagnostics and Therapeutics, Joint Faculty of Veterinary Medicine, Yamaguchi University, Yamaguchi, Japan.

**Keywords:** mouse-canine chimeric antibody, dog podoplanin, dPDPN, monoclonal antibody

## Abstract

Podoplanin (PDPN), a type I transmembrane sialoglycoprotein, is expressed in normal tissues, including lymphatic endothelial cells, pulmonary type I alveolar cells, and renal podocytes. The overexpression of PDPN in cancers is associated with hematogenous metastasis by interactions with the C-type lectin-like receptor 2 (CLEC-2). We have previously reported the development of a mouse monoclonal antibody (mAb) clone, PMab-38 (IgG_1_, kappa), against dog PDPN (dPDPN). PMab-38 reacted strongly with canine squamous cell carcinomas and melanomas, but not with lymphatic endothelial cells, indicating its cancer specificity. In this study, we developed and produced several mouse-canine chimeric antibodies originating from PMab-38. A mouse-canine chimeric antibody of subclass A (P38A) and a mouse-canine chimeric antibody of subclass B (P38B) were transiently produced using ExpiCHO-S cells. Core-fucose-deficient P38B (P38Bf) was developed using FUT8 knockout ExpiCHO-S cells. We compared the binding affinities, antibody-dependent cellular cytotoxicity (ADCC), and complement-dependent cytotoxicity (CDC) of P38A, P38B, and P38Bf against Chinese hamster ovary (CHO)/dPDPN cells. Flow cytometry analysis showed that the *K*_D_ of P38A, P38B, and P38Bf were 1.9 × 10^−7^, 5.2 × 10^−9^, and 6.5 × 10^−9^, respectively. Both P38B and P38Bf revealed high ADCC activities against CHO/dPDPN cells; P38Bf demonstrated significantly higher ADCC compared with P38B, especially at low concentrations. P38B and P38Bf exhibited higher CDC activities against CHO/dPDPN cells. Conversely, P38A did not exhibit any ADCC or CDC activity. In summary, P38Bf is a good candidate for antibody therapy against dPDPN-expressing canine cancers.

## Introduction

Podoplanin (PDPN) is known to be expressed in normal tissues, including lymphatic endothelial cells, pulmonary type I alveolar cells, renal podocytes, chondrocytes, myofibroblasts, and mesothelial cells.^(1)^ An elevated expression of PDPN is also observed in different types of tumors, such as squamous cell carcinomas (SCCs),^(2)^ testicular tumors,^(3)^ glioblastoma,^(4)^ and mesothelioma.^(5,6)^ Recent clinical studies have provided evidence for the association between increased PDPN expression and poor disease prognosis,^(7)^ indicating that the establishment of anti-PDPN monoclonal antibodies (mAbs) is critical for developing novel therapeutic strategies against cancer development and metastatic progression.^(8)^

Dog PDPN (dPDPN) was previously reported as gp40.^(9)^ We developed two mAbs namely, PMab-38 (mouse IgG_1_, kappa)^(10)^ and PMab-48 (mouse IgG_1_, kappa),^(11)^ which specifically recognize dPDPN. PMab-38 recognized dPDPN of renal epithelial cells, but did not react with lymphatic endothelial cells.^(10)^ Conversely, PMab-48 reacted not only with renal epithelial cells but also with lymphatic endothelial cells.^(11)^ Tyr67 and Glu68 of dPDPN were determined as the critical epitopes of PMab-38.^(12)^ Contrastingly, Asp29, Asp30, Ile31, Ile32, and Pro33 of dPDPN were found to be necessary for recognition of PMab-48.^(13)^ Using immunohistochemistry, we further demonstrated that PMab-38 reacted with 83% of canine SCCs (15/18 cases)^(14)^ and 90% of melanomas (9/10 cases),^(15)^ indicating that PMab-38 is applicable for antibody-based therapy for canine cancers. In this study, we produced several mouse-canine chimeric antibodies from PMab-38 and investigated their antibody-dependent cellular cytotoxicity (ADCC) and complement-dependent cytotoxicity (CDC) activities.

## Materials and Methods

### Cell lines

Chinese hamster ovary (CHO)-K1 cell line was obtained from the American Type Culture Collection (ATCC, Manassas, VA). In our previous studies, we inserted dPDPN with an N-terminal PA tag and a C-terminal RAP tag-MAP tag (PA-dPDPN-RAP-MAP) in a pCAG-Ble vector (FUJIFILM Wako Pure Chemical Corporation, Osaka, Japan).^(10)^ The PA tag,^(16)^ RAP tag,^(17)^ and MAP tag^(18)^ consist of 12 amino acids each, namely, GVAMPGAEDDVV, DMVNPGLEDRIE, and GDGMVPPGIEDK, respectively. CHO-K1 cells were transfected with pCAG-Ble/PA-dPDPN-RAP-MAP using Gene Pulser Xcell electroporation system (Bio-Rad Laboratories, Inc., Berkeley, CA) resulting in the cell line CHO/dPDPN. CHO-K1 and CHO/dPDPN were cultured in RPMI 1640 medium (Nacalai Tesque, Inc., Kyoto, Japan) supplemented with 10% heat-inactivated fetal bovine serum (Thermo Fisher Scientific, Inc., Waltham, MA), 100 units/mL of penicillin, 100 μg/mL of streptomycin, and 25 μg/mL of amphotericin B (Nacalai Tesque, Inc.) at 37°C in a humidified atmosphere of 5% CO_2_ and 95% air.

### Antibodies

PMab-38, a mouse anti-dPDPN mAb (IgG_1_, kappa), was developed as previously described.^(10)^ To generate a mouse-canine (subclass A) chimeric antibody, P38A, the appropriate V_H_ and V_L_ cDNAs of mouse PMab-38 and the C_H_ and C_L_ of canine IgG subclass A were subcloned into pCAG-Ble and pCAG-Neo vectors (FUJIFILM Wako Pure Chemical Corporation), respectively. Similarly, to generate a mouse-canine (subclass B) chimeric antibody, P38B, the appropriate V_H_ and V_L_ cDNAs of mouse PMab-38 and the C_H_ and C_L_ of canine IgG subclass B were subcloned into pCAG-Ble and pCAG-Neo vectors (FUJIFILM Wako Pure Chemical Corporation), respectively. To express P38A and P38B, antibody expression vectors were transfected into ExpiCHO-S cells using the ExpiFectamine CHO Transfection kit (Thermo Fisher Scientific, Inc.). To generate P38Bf, antibody expression vectors were transfected into BINDS-09 (FUT8-knocked out ExpiCHO-S cells^*^) using the ExpiFectamine CHO Transfection kit. P38A, P38B, and P38Bf were purified using Protein G-Sepharose (GE Healthcare Bio-Sciences, Pittsburgh, PA).

### Flow cytometry

Cells were harvested after brief exposure to 0.25% trypsin/1 mM ethylenediaminetetraacetic acid (Nacalai Tesque, Inc.). After washing with 0.1% bovine serum albumin in phosphate-buffered saline, the cells were treated with P38A, P38B, and P38Bf (0.1–10 μg/mL) for 30 minutes at 4°C, followed by treatment with FITC-conjugated anti-dog IgG (1:200; Sigma-Aldrich Corp., St. Louis, MO). Fluorescence data were acquired using the Cell Analyzer EC800 (Sony Corp., Tokyo, Japan).

### Determination of binding affinity using flow cytometry

CHO/dPDPN cells (2 × 10^5^) were resuspended in 90 μL of serially diluted P38A, P38B, and P38Bf (6 ng/mL to 100 μg/mL), followed by the addition of secondary anti-dog IgG (1:30; Sigma-Aldrich Corp., St. Louis, MO). Fluorescence data were collected using a cell analyzer (EC800). *K*_D_ was obtained by fitting the binding isotherms using the built-in one-site binding models in GraphPad PRISM 6 (GraphPad Software, La Jolla, CA).

### Antibody-dependent cellular cytotoxicity

Canine lymphokine-activated killer (LAK) cells, used as effector cells, were prepared from peripheral blood mononuclear cells from a healthy beagle dog by culturing in the presence of 1000 IU/mL of human recombinant IL-2 for 1 week. CHO-K1/luc and CHO/dPDPN/luc cells were used as target cells.^(19)^ Both cells were generated by transducing the luciferase reporter vector into CHO-K1 and CHO/dPDPN cells. CHO-K1/luc cells and CHO/dPDPN/luc cells (5 × 10^3^) were seeded into 96-well flat-bottom microtiter plates containing dog IgG whole molecule (Jackson ImmunoResearch, Inc., PA), P38A, P38B, or P38Bf. After incubating on ice for 15 minutes, the LAK cells were added at an effector/target ratio of 20:1. For maximal killing control, 2% Triton X-100 was added. Cultures were incubated further for 4 hours followed by cell lysis using the ONE-Glo Luciferase assay system (Promega, Madison, WI) according to manufacturer's instructions. Luciferase activities were detected by the ARVO X4 system (PerkinElmer, Waltham, MA). ADCC was calculated as follows: duplicate wells were averaged and percent lysis was calculated from the data using the following equation: % specific lysis = 100 × (spontaneous death RLU − test RLU)/(spontaneous death RLU − maximal killing RLU). Experiments were repeated thrice and results are shown as a mean of three independent experiments.

### Complement-dependent cytotoxicity

Luciferase-expressing CHO-K1 cells and CHO/dPDPN cells (5 × 10^3^) were seeded into 96-well round-bottom microtiter plates containing either whole molecule dog IgG, P38A, P38B, or P38Bf. After incubating on ice for 20 minutes, LOW-TOX^®^-H rabbit complement at a dilution of 1:40 (CEDALANE, Ontario, Canada) was added to each well and incubated further for 90 minutes. This was followed by cell lysis using the ONE-Glo Luciferase assay system (Promega) according to the manufacturer's instructions. Luciferase activities were detected by the ARVO X4 system (PerkinElmer). CDC was calculated as follows: duplicate wells were averaged and percent lysis was calculated from the data using the following equation: % specific lysis = 100 × (spontaneous death RLU − test RLU)/(spontaneous death RLU − maximal killing RLU). The experiments were repeated thrice and results are shown as an average of three independent experiments.

## Results and Discussion

Previously, we developed a mouse anti-dPDPN mAb, namely, PMab-38, by immunization using dPDPN proteins, which were expressed in CHO-K1 cells.^(10)^ PMab-38 exhibits a high sensitivity and specificity against dPDPN and is highly suitable for the detection of PDPN-expressing cancer cells of SCCs and melanomas in immunohistochemical analyses.^(14,15)^ Conversely, PMab-38 reacts weakly with podocytes of the canine kidney and does not recognize lymphatic endothelial cells, indicating that PMab-38 is a cancer-specific mAb. We previously established cancer-specific mAbs (CasMabs) against human PDPN, such as LpMab-2^(8)^ and LpMab-23.^(20)^ These cancer-specific mAbs may prove advantageous for targeting cancer cells without adverse effects.

Few studies have already been performed concerning the subclasses (A, B, C, and D) of canine IgGs.^(21,22)^ Bergeron et al. clearly showed that the canine subclasses A and D appear effector-function negative, while the subclasses B and C bind canine Fc gamma receptors and are positive for antibody-dependent cellular cytotoxicity (ADCC) similar to human IgG_1_ and IgG_3_, respectively.^(21)^ Furthermore, the subclasses B and C can induce CDC. Rue et al. established an anti-canine CD20 mAb (1E4) to treat canine B-cell lymphoma and produced mouse-canine chimeric antibodies.^(22)^ They also showed that 1E4-cIgGB (subclass B) and 1E4-cIgGC (subclass C) led to significant depletion of B-cell levels in healthy beagle dogs.

**FIG. 1.**
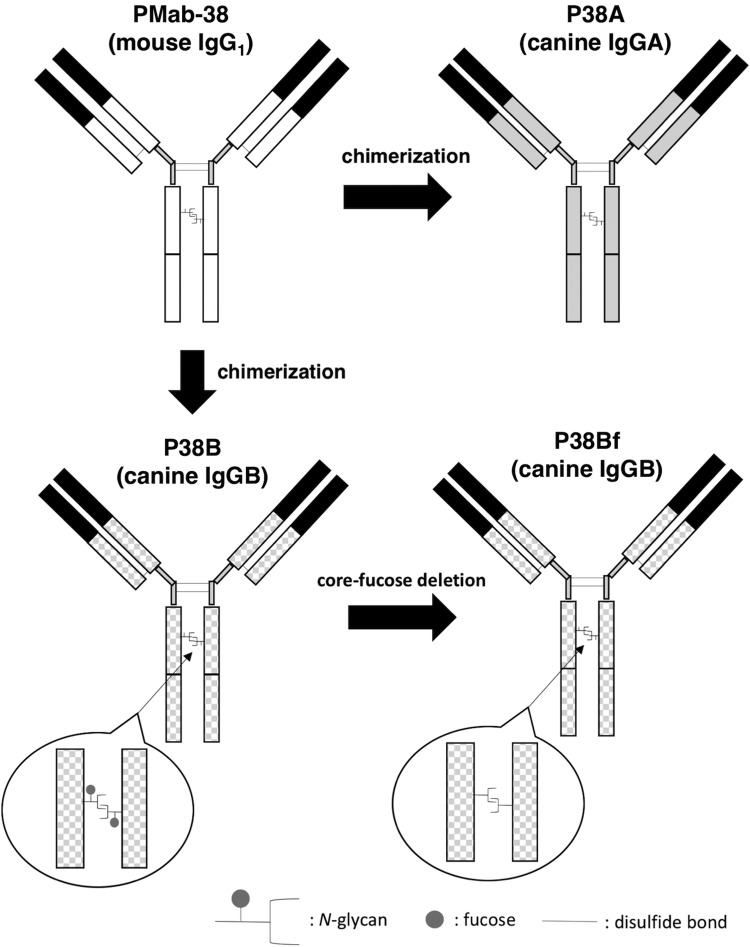
Production of P38A (canine IgGA), P38B (canine IgGB), and P38Bf (canine IgGB; core-fucose deficient) from PMab-38 (mouse IgG_1_).

**FIG. 2.**
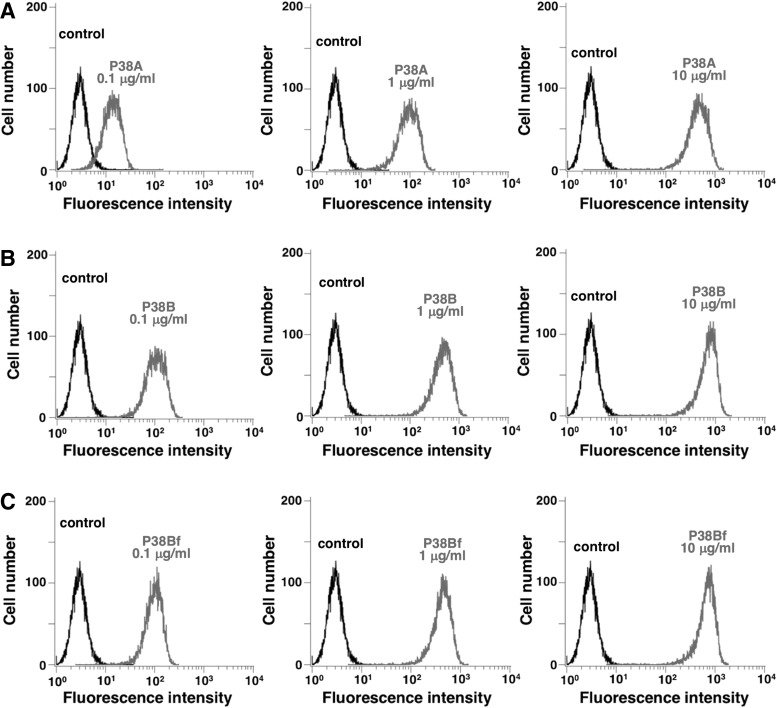
Flow cytometry. CHO/dPDPN cells were treated with **(A)** P38A, **(B)** P38B, and **(C)** P38Bf, followed by treatment with FITC-conjugated anti-canine IgG. Fluorescence data were collected using a cell analyzer. CHO, Chinese hamster ovary; dPDPN, dog podoplanin.

**FIG. 3.**
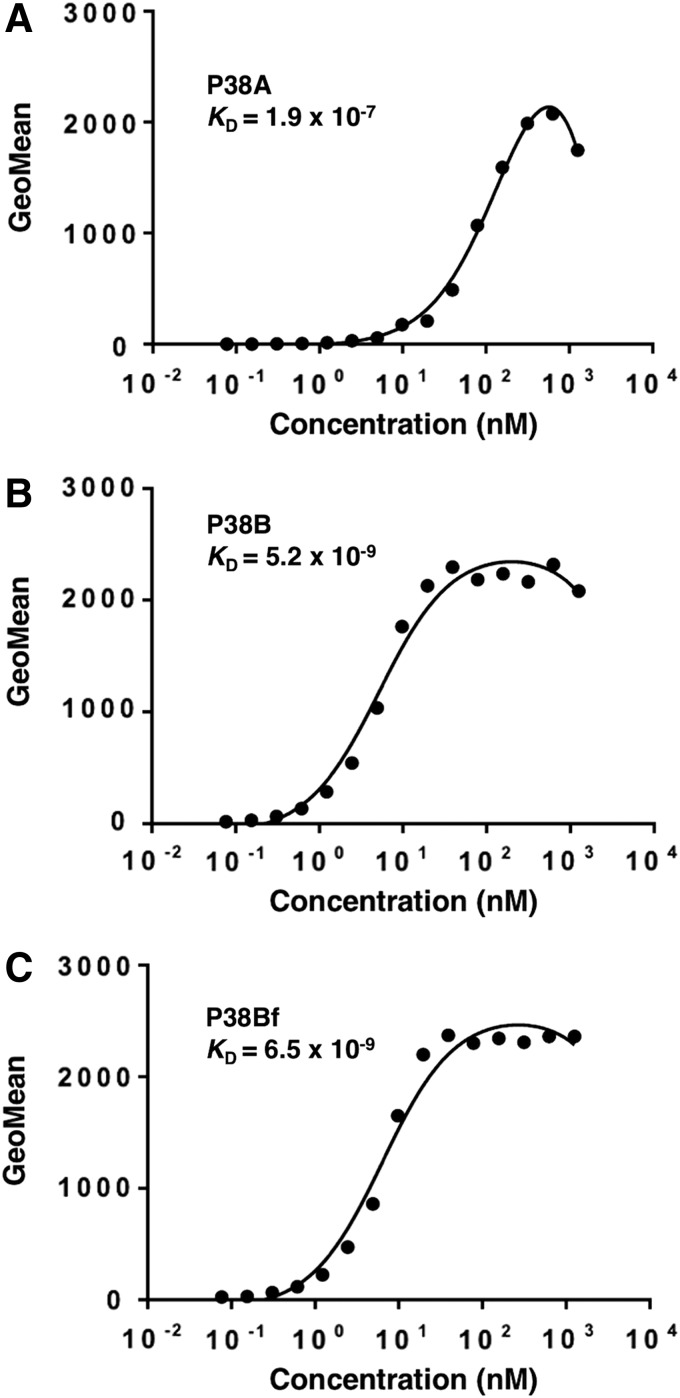
Determination of binding affinities using flow cytometry. CHO/dPDPN cells were suspended in 90 μL of serially diluted antibodies (6 ng/mL to 100 μg/mL): **(A)** P38A, **(B)** P38B, and **(C)** P38Bf, followed by treatment with FITC-conjugated anti-canine IgG. Fluorescence data were collected using a cell analyzer. GeoMean, geometric mean.

In this study, we first developed several mouse-canine chimeric antibodies by combining the variable regions of PMab-38 with the constant regions of different canine IgG subclasses (Fig. 1). A canine IgG subclass A corresponding to the human IgG_2_ does not possess ADCC and CDC activities.^(21)^ In contrast, a canine IgG subclass B corresponding to human IgG_1_ possesses high ADCC and CDC activities. Furthermore, deletion of core-fucose from the human IgG_1_ has been reported to induce significantly higher ADCC activity.^(23)^ Therefore, we produced a core-fucose-deficient mouse-canine chimeric antibody using FUT8 knockout cell lines. Resultantly, P38A (subclass A), P38B (subclass B), and P38Bf (core-fucose-deficient subclass B) were developed. As shown in Figure 2, three chimeric antibodies showed high sensitivity against CHO/dPDPN cells. Among these, P38B and P38Bf showed a significantly higher reaction compared with P38A at low concentrations suggesting that (i) the constant regions of the subclass B might be more stable than those of subclass A in this study and (ii) the depletion of core-fucose from canine IgG might not reduce sensitivity of the antibody. We next compared the binding affinity of P38A, P38B, and P38Bf and the *K*_D_ of P38A, P38B, and P38Bf was determined to be 1.9 × 10^−7^, 5.2 × 10^−9^, and 6.5 × 10^−9^, respectively, using flow cytometry analysis (Fig. 3). This clearly indicated a higher binding affinity of P38B and P38Bf compared with P38A. In this study, we utilized the constant regions of canine IgGs, which were obtained commercially. We aim to further investigate the binding affinities of canine IgGs possessing different constant regions obtained from other sources, to clearly discuss the binding affinities and stability of various canine IgGs.

**FIG. 4.**
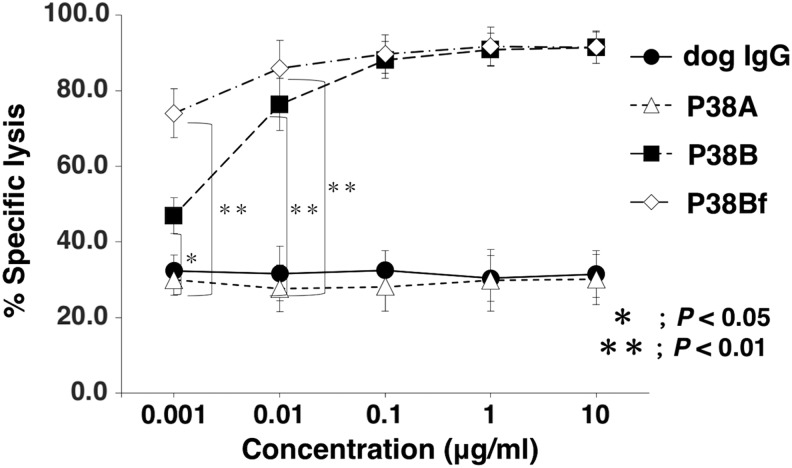
ADCC activity of P38A, P38B, and P38Bf against dPDPN-expressing CHO cells. ADCC was determined by luciferase-based cytotoxicity assay. CHO/dPDPN/luc cells were incubated with serially diluted dog IgG, P38A, P38B, and P38Bf antibodies (0.001–10 μg/mL) in the presence of LAK cells at an effector: target ratio of 20:1 for 4 hours. Luciferase activity was measured by lysis of live cells after appropriate incubation time periods. Values presented are mean ± SEM of three independent experiments. *P < 0.05, **P < 0.01, Tukey–Kramer's test. ADCC, antibody-dependent cellular cytotoxicity; LAK, lymphokine-activated killer; SEM, standard error of the mean.

We next investigated ADCC activities using P38A, P38B, and P38Bf. To this end, we used CHO/dPDPN/luc as target cells and LAK cells as effector cells. Both P38B and P38Bf revealed high ADCC activities against CHO/dPDPN/luc cells; P38Bf demonstrated significantly higher ADCC than P38B, especially at low concentrations such as 1 ng/mL (Fig. 4). Both P38B and P38Bf did not exhibit ADCC against parental CHO-K1/luc cells (Supplementary Fig. S1), indicating that cytotoxicity is dependent on dPDPN overexpressed in CHO-K1 cells. Conversely, P38A did not exhibit ADCC activity against CHO/dPDPN/luc cells (Fig. 4), confirming that the subclass A of canine IgG could not induce ADCC.

**FIG. 5.**
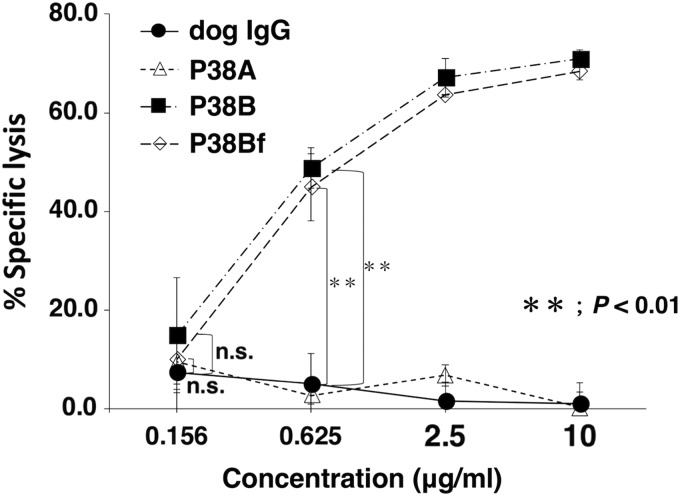
CDC activity of P38A, P38B, and P38Bf against dPDPN-expressing CHO cells. CDC was determined by luciferase-based cytotoxicity assay. CHO/dPDPN/luc cells were incubated with a rabbit complement at a dilution of 1:40 in the presence of serially diluted dog IgG, P38A, P38B, and P38f antibodies (0.156–10 μg/mL) for 90 minutes in 96-well plates. Luciferase activity was measured after lysis of live cells. Values presented are mean ± SEM of three independent experiments. **P < 0.01, Tukey–Kramer's test. CDC, complement-dependent cytotoxicity; n.s., not significant.

Furthermore, we investigated CDC activities using P38A, P38B, and P38Bf by using CHO/dPDPN/luc as target cells. As shown in Figure 5, P38B and P38Bf showed high CDC activities against CHO/dPDPN cells. Both P38B and P38Bf did not show CDC against parental CHO-K1/luc cells (Supplementary Fig. S2), indicating that P38B- and P38Bf-mediated CDC are dependent on dPDPN overexpression in CHO-K1 cells. Contrastingly, P38A did not exhibit any CDC activity (Fig. 5), which confirmed that the subclass A of canine IgG could not induce CDC.

Taken together, P38Bf is applicable for antibody therapy against canine cancers expressing dPDPN. Further studies on ADCC and CDC are necessary to obtain a more detailed understanding of endogenous dPDPN-expressing canine cancer cells.

## Supplementary Material

Supplemental data

Supplemental data

## Notes

^*^ www.med-tohoku-antibody.com/topics/001_paper_cell.htm
