# Supplementary material for: Establishment of P38Bf, a Core-Fucose-Deficient Mouse-Canine Chimeric Antibody Against Dog Podoplanin
Source: Monoclon Antib Immunodiagn Immunother. 2018 Oct 24;37(5):218–23. doi: 10.1089/mab.2018.0035 (PMC6208159; doi:10.1089/mab.2018.0035)
Supplement: Supplemental data [file Supp_Fig1.pdf]

Supplementary Data

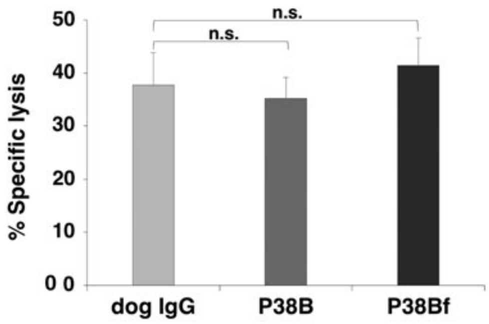

SUPPLEMENTARY FIG. S1. ADCC activity of P38B, and P38Bf against CHO-K1/luc cells. ADCC against parental luciferase-expressing CHO-K1 cells was determined as described in Figure 4. ADCC, antibody-dependent cellular cytotoxicity; CHO, Chinese hamster ovary; n.s., not significant.
