# Supplementary material for: Establishment of P38Bf, a Core-Fucose-Deficient Mouse-Canine Chimeric Antibody Against Dog Podoplanin
Source: Monoclon Antib Immunodiagn Immunother. 2018 Oct 24;37(5):218–23. doi: 10.1089/mab.2018.0035 (PMC6208159; doi:10.1089/mab.2018.0035)
Supplement: Supplemental data [file Supp_Fig2.pdf]

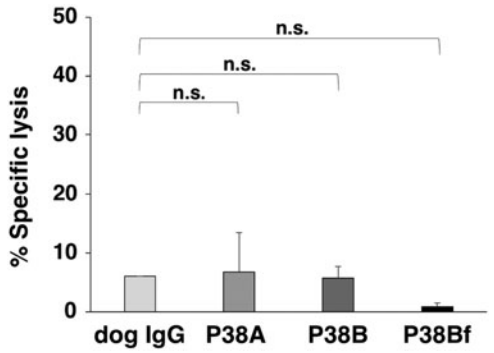

SUPPLEMENTARY FIG. S2. CDC activity of P38A, P38B, and P38Bf against CHO-K1/luc cells. CDC against parental luciferase-expressing CHO-K1 cells was determined as described in Figure 5. CDC, complement-dependent cytotoxicity.
